# False-positive results for seasonal coronavirus infections on using the FilmArray Pneumonia Panel

**DOI:** 10.1128/spectrum.01343-24

**Published:** 2024-10-29

**Authors:** Kuenyoul Park, Bosung Park, Eun Jeong Won, Heungsup Sung, Mi-Na Kim

**Affiliations:** 1 Department of Laboratory Medicine, Sanggye Paik Hospital, School of Medicine, Inje University, Seoul, South Korea; 2 Department of Laboratory Medicine, Asan Medical Center, University of Ulsan College of Medicine, Seoul, South Korea; MultiCare Health System, Tacoma, Washington, USA

**Keywords:** pneumonia, coronavirus, false-positive, cross-reactivity, melting temperature

## Abstract

The FilmArray Pneumonia Panel (FilmArrayPN; bioMérieux) was tagged for potential false-positive seasonal coronavirus results, possibly caused by non-specific amplification or cross-reactivity with human genomic DNA. This study was conducted to determine the real-world false-positive rate of seasonal coronavirus infections using FilmArrayPN at a tertiary care hospital. We retrospectively reviewed FilmArrayPN results to detect lower respiratory tract pathogens from July 2023 to April 2024. Allplex Respiratory Panel (AllplexRP; Seegene) and/or FilmArray Respiratory Panel (FilmArrayRP) results for the same patient were considered as reference results. In the last month of the study period, all positive samples in FilmArrayPN were confirmed by AllplexRP and pan-coronavirus targeted PCR (pan-CoV PCR). A total of 2,120 specimens, including 1,847 expectorated sputum and 273 bronchial aspirate (BA) samples, were tested by FilmArrayPN. Of these, 161 (8.7%) sputum and 7 (2.6%) BA specimens from 152 patients were positive for coronavirus targets in FilmArrayPN. Of the 122 cases also tested by AllplexRP and/or FilmArrayRP, 106 specimens (86.9%) were coronavirus-negative, except for 9 OC43-positive, 6 229E-positive, and 1 NL63-positive. Notably, 83.1% of the 106 cases that tested negative in the other tests had melting temperatures above 83℃. A total of 61 specimens that tested positive for coronavirus in FilmArrayPN but negative in AllplexRP were confirmed to be negative in pan-CoV PCR. The coronavirus positivity of 7.8% in the FilmArrayPN resulted in 5% of samples being potentially misreported as false-positives. This report thus highlights the need for continuous monitoring of melting temperatures to avoid potential false-positives.

**IMPORTANCE:**

The FilmArray Pneumonia Panel (FilmArrayPN; bioMérieux) was tagged for potential false-positive seasonal coronavirus results, possibly caused by non-specific amplification or cross-reactivity with human genomic DNA. FilmArrayPN results were retrospectively reviewed from July 2023 to May 2024. Of 2,120 tested specimens, 168 specimens from 152 patients were positive for coronavirus targets in FilmArrayPN. Of the 122 cases also tested by Allplex Respiratory Panel and/or FilmArray Respiratory Panel, 106 specimens (86.9%) were coronavirus-negative. Notably, 83.1% of the 106 cases that tested negative in the other tests had melting temperatures above 83℃. A total of 61 specimens that tested positive for coronavirus in FilmArrayPN but negative in Allplex were confirmed to be negative in pan-coronavirus targeted PCR. The coronavirus positivity of 7.8% in the FilmArrayPN resulted in 5% of samples being potentially misreported as false-positives. This report highlights the need for continuous monitoring of melting temperatures to avoid potential false-positives.

## OBSERVATION

The FilmArray Pneumonia Panel (FilmArrayPN; bioMérieux) is a robust tool for detecting pathogens, including viruses and atypical bacteria (1), detecting multidrug resistance (2), and influencing antimicrobial stewardship (3). The FilmArrayPN was tagged in South Korea in February 2024 on account of potential false-positive seasonal coronavirus results likely caused by non-specific amplification or cross-reactivity with human genomic DNA. Since 2014, the manufacturer has reported false-positive results from the FilmArray BCID and BCID2 panels due to contamination of blood culture bottles with organisms such as *Pseudomonas aeruginosa*, *Enterococcus* spp., *Proteus* spp., *Escherichia coli*, *Acinetobacter calcoaceticus-baumannii* complex, and *Candida tropicalis* (4), which has also been noted in updates on the U.S. Food and Drug Administration website. A recent meta-analysis revealed 4–11% false-positives using the meningitis/encephalitis panel (5), and multicenter FilmArrayPN evaluations reported 88.9% specificity for *Haemophilus influenzae* (6). The present study aimed to determine the real-world false-positive rate of seasonal coronavirus infections using FilmArrayPN at a tertiary care hospital as the actual proportion of false-positive results remains unknown. The study involved reviewing and comparing FilmArrayPN results for the detection of lower respiratory tract pathogens for 10 months beginning from July 2023 and confirmatory testing for seasonal coronavirus in March 2024.

We analyzed FilmArrayPN results for the detection of lower respiratory tract pathogens for 10 months beginning from July 2023 at a 2,764-bed tertiary care hospital in Seoul, South Korea, which approved FilmArrayPN testing in expectorated sputum and bronchial aspirate (BA) from July 2023. Samples were collected in 50-mL conical tubes and sent to the central clinical microbiology laboratory. All FilmArrayPN results were generally reported within 2 h. Subtypes of seasonal coronaviruses were not identified in FilmArrayPN. Following an alert from the manufacturer in February 2024, all FilmArrayPN-positive coronavirus results were confirmed using the Allplex Respiratory Panel (AllplexRP; Seegene) from March 2024 onward. FilmArrayPN detects all four seasonal coronavirus strains (229E, OC43, NL63, and HKU1), whereas AllplexRP detects coronavirus 229E, OC43, and NL63, but not HKU1. Manufacturer-claimed limits of detection of the FilmArrayPN differed from 81 to 10,000 copies/mL, depending on the subtype (229E: 81, NL63: 540, OC43: 9300, and HKU1: 10,000 copies/mL). In contrast, those of the AllplexRP were claimed to be 100 copies/mL for the majority of the viruses, including coronavirus. For confirming false-positivity for four subtypes of coronaviruses, samples that tested positive for coronaviruses obtained from March 20, 2024, onward were subjected to pan-coronavirus targeted PCR (pan-CoV PCR), the limit of detection of which was not established. The protocol used for pan-CoV PCR was as follows: reactions (using 25 µL mixtures) were performed using the one-step RT-PCR kit from QIAGEN (Hilden, Germany) with 200 nM primer PC2S2 (equimolar mixture of TTATGGGTTGGGATTATC and TGATGGGGATGGGACTATC), 900 nM primer PC2As1 (equimolar mixture of TCATCACTCAGAATCATCA, TCATCAGAAAGAATCATCA, and TCGTCGGACAAGATCATCA), 1 µL QIAGEN one-step RT-PCR kit enzyme mix, and 5 µL RNA extract (7).

Results of the AllplexRP and FilmArray Respiratory Panel (FilmArrayRP; bioMérieux) were retrieved from electronic medical records for all specimens that tested positive in FilmArrayPN during the study period. These results were considered reference results, and FilmArrayPN-only positive cases were regarded as possible false-positives because the AllplexRP panel cannot confirm negativity for HKU1. FilmArrayPN coronavirus-positive cases from before March 2024 were retrospectively reviewed for potential false-positives if the patients had been concurrently tested with either AllplexRP or FilmArrayRP. Following a manufacturer’s alert in February 2024, all FilmArrayPN coronavirus-positive cases were prospectively confirmed using the AllplexRP panel. Cases that tested negative in pan-CoV PCR were confirmed as false-positives. Furthermore, all coronavirus-positive cases on FilmArrayPN were manually confirmed using amplification curves and melting temperatures for the coronavirus targets. The melting temperatures were plotted along with reference results using R package ggplot2 v.3.5.1. MedCalc (version 20.305; MedCalc Software, Ostend, Belgium) was used to calculate 95% confidence intervals (CIs) of proportions and perform the Mann–Whitney test for comparison. This study was approved by the Institutional Review Board of Asan Medical Center, Seoul, and the requirement of obtaining informed consent from patients was waived (Institutional Review Board no. 2024-0505).

A total of 2,120 specimens, including 1,847 expectorated sputum and 273 BAs, were tested. Of these, 161 (8.7%; 95% CI, 7.5–10.1%) sputum and 7 (2.6%; 95% CI, 1.0–5.2%) BA specimens from 152 patients were positive for coronavirus in FilmArrayPN. AllplexRP or FilmArrayRP testing performed concurrently on 122 specimens, including 119 sputum specimens and 3 BAs, revealed 106 possible false-positives (86.9%; 95% CI, 79.6–92.3%), including 103 sputum specimens and 3 BAs. AllplexRP identified 16 true-positive cases, including 9 OC43-positive sputum specimens with cycle threshold (Ct) values of 16.84–38.22 (median, 30.24), 6 229E-positive sputum specimens with Ct values of 20.56–37.23 (median, 30.26), and 1 NL63-positive sputum specimen with a Ct value of 27.36. In addition, FilmArrayRP detected one OC43-positive case. Using pan-CoV PCR for specimens submitted in the last month of the study period, all 61 specimens positive for coronavirus infection in FilmArrayPN but negative in AllplexRP were confirmed as false-positives. We confirmed that all 12 samples that tested positive for CoV on the FilmArrayPN and had a Ct value of ≤33.12 on the AllplexRP were also positive in pan-CoV PCR. In contrast, the four remaining samples—two OC43 samples with Ct values of 38.22 and 37.6 and two 229E samples with Ct values of 37.23 and 36.61—tested negative in pan-CoV PCR.

**FIG 1:**
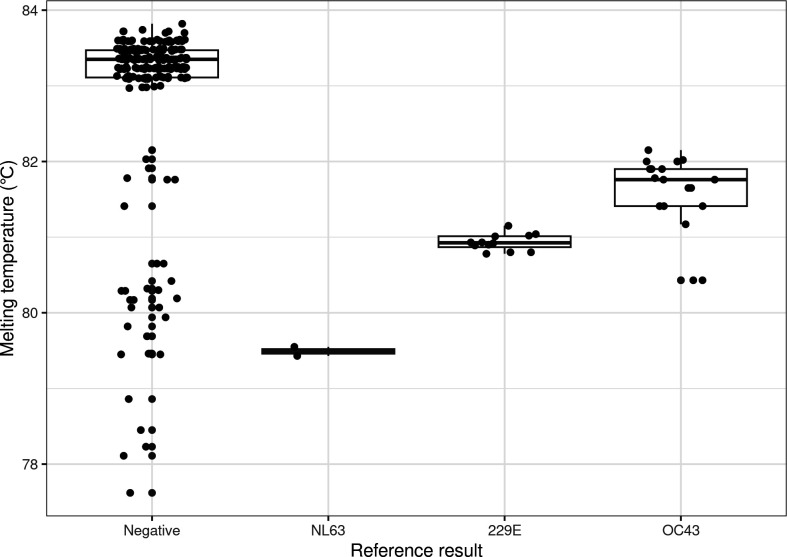
Melting temperature distribution of coronavirus targets from the FilmArray Pneumonia Panel (bioMérieux) along with reference results from the Allplex Respiratory Panel (Seegene) and FilmArray Respiratory Panel (bioMérieux) performed using the samples collected at the same visits. Two melting temperature points from duplicate coronavirus wells were observed from a case that tested positive on the FilmArray Pneumonia Panel.

The median values of melting temperatures of coronavirus targets differed by coronavirus type: 229E (80.9), NL63 (79.5), and OC43 (81.8). The possible false-positives had a higher median value (83.3°C) than true positives (81.3°C) (*P* < 0.001). Notably, 2.6% and 83.1% of the 106 possible false-positives had melting temperatures below 79°C and above 83°C, respectively, which was not evident in true positives (Fig. 1).

We retrospectively reviewed 2,120 tests performed using the FilmArrayPN for 168 cases (7.9%) that tested positive for coronavirus infections. Of these, 86.9% (106/122) were considered possible false-positives, resulting in a total possible false-positivity of 5.0% (106/2120). None of the pan-CoV PCR-positive cases were HKU1. The FilmArrayRP was primarily used in pediatric patients, a patient population different from that tested with the FilmArrayPN panel, during the study period. FilmArrayRP results showed that HKU1 was detected in 0.6% of cases, OC43 in 2.8%, NL63 in 1.8%, and 229E in 0.3% (unpublished data). The absence of HKU1 in the AllplexRP results likely represents a minority of potential false-positive cases. A previous study reported only a single sample positive for coronavirus infection in FilmArrayPN that was simultaneously negative for the reference test among 200 specimens in the US (8). False-positive rates might be affected by carry-over contamination during sample collection or testing, especially when infection rates are high. However, in our review of test results, we have rarely observed false-positive results due to carry-over contamination from true-positive samples. Furthermore, FilmArray panels are susceptible to false-positives because of the use of a fluorescent double-stranded DNA-binding dye (9). Manual interpretation enabled ruling out false-positives while using the FilmArrayRP during the COVID-19 pandemic because non-specific amplification observed in false-positive cases was often observed in one well rather than both wells targeting the same pathogen (10). However, we observed positive amplification in both wells in FilmArrayPN for all coronavirus-positive cases. Thus, despite a manual review of amplification curves, 5% of cases tested for coronavirus infection using the FilmArrayPN, all of which were positive in both wells, may have been falsely reported as positive.

While more than 80% of possible false-positive cases had melting temperatures above 83°C, the same was not observed for any of the confirmed positive cases. Additionally, since a positive result for coronavirus HKU1 had never been obtained on a pneumonia panel during this period, one specimen positive for the HKU1 target in the FilmArrayRP was tested using the FilmArrayPN and was found to have a melting temperature of 79.6°C for the coronavirus target (unpublished data). Melting temperature analysis is used to differentiate amplification products (11) and/or exclude false-positive cases (12). As per the manufacturer’s warning, these false-positives might be a consequence of cross-reactivity with high concentrations of human genomic DNA. Accordingly, amplicons with melting temperatures above 83°C observed in possible false-positives could have originated from human genomic DNA. Thus, the melting temperature range for coronavirus targets in the FilmArrayPN requires modification, and amplification curves as well as melting temperatures need to be continuously monitored to exclude false-positives.

This study has a few limitations. First, the lack of confirmatory testing for most positive specimens. However, results from other panels using respiratory specimens were used as reference results and a few cases were confirmed using pan-CoV PCR. Second, the concentration of human DNA, which may result in false-positive results, may differ between sputum samples. Thus, real-world false-positive rates may vary in different patient populations. Third, the pan-CoV PCR assay may have been limited in resolving discrepancies between the FilmArrayPN and the AllplexRP assay because we did not determine the limit of detection for the pan-CoV PCR, which may have resulted in lower sensitivity compared with the AllplexRP assay, particularly for samples with a Ct value >33.12. However, given the broad detection capability across coronaviruses of the pan-CoV PCR and the relatively low reported limit of detection of FilmArrayPN compared with the AllplexRP assay, pan-CoV negativity in AllplexRP-negative and FilmArrayPN-positive cases appears to indicate a false-positive caused by non-specific amplification. Lastly, differences in analytical sensitivity between panels may affect some discrepant cases with FilmArrayPN-only positives. However, the claimed limit of detection of the AllplexRP panel is similar to or even lower than that of FilmArrayPN, which may not explain the higher positivity rate of the FilmArrayPN in this study.

In conclusion, 5% of samples assayed using the FilmArrayPN were potentially misreported as false-positives, considering a coronavirus positivity rate of 7.8% in the FilmArrayPN. This finding highlights the need for continuous monitoring of melting temperatures along with manual interpretation of amplification curves to avoid potential false-positive results.
